# Identification of the prognostic signature based on genomic instability-related alternative splicing in colorectal cancer and its regulatory network

**DOI:** 10.3389/fbioe.2022.841034

**Published:** 2022-07-18

**Authors:** Qiuying Ding, Zhengping Hou, Zhibo Zhao, Yao Chen, Lei Zhao, Yue Xiang

**Affiliations:** ^1^ Centre for Lipid Research, Key Laboratory of Molecular Biology for Infectious Diseases, Ministry of Education, Department of Infectious Diseases, Institute for Viral Hepatitis, The Second Affiliated Hospital, Chongqing Medical University, Chongqing, China; ^2^ The Department of Hepatobiliary Surgery of the Second Affiliated Hospital, Chongqing Medical University, Chongqing, China

**Keywords:** colorectal cancer, genomic instability, alternative splicing, splicing factor, overall survival

## Abstract

**Background:** Colorectal cancer (CRC) is a heterogeneous disease with many somatic mutations defining its genomic instability. Alternative Splicing (AS) events, are essential for maintaining genomic instability. However, the role of genomic instability-related AS events in CRC has not been investigated.

**Methods:** From The Cancer Genome Atlas (TCGA) program, we obtained the splicing profiles, the single nucleotide polymorphism, transcriptomics, and clinical information of CRC. Combining somatic mutation and AS events data, a genomic instability-related AS signature was constructed for CRC. Mutations analyses, clinical stratification analyses, and multivariate Cox regression analyses evaluated this signature in training set. Subsequently, we validated the sensitivity and specificity of this prognostic signature using a test set and the entire TCGA dataset. We constructed a nomogram for the prognosis prediction of CRC patients. Differentially infiltrating immune cells were screened by using CIBERSORT. Inmmunophenoscore (IPS) analysis was used to evaluate the response of immunotherapy. The AS events-related splicing factors (SF) were analyzed by Pearson’s correlation. The effects of SF regulating the prognostic AS events in proliferation and migration were validated in Caco2 cells.

**Results:** A prognostic signature consisting of seven AS events (PDHA1-88633-ES, KIAA1522-1632-AP, TATDN1-85088-ES, PRMT1-51042-ES, VEZT-23786-ES, AIG1-77972-AT, and PHF11-25891-AP) was constructed. Patients in the high-risk score group showed a higher somatic mutation. The genomic instability risk score was an independent variable associated with overall survival (OS), with a hazard ratio of a risk score of 1.537. The area under the curve of receiver operator characteristic curve of the genomic instability risk score in predicting the OS of CRC patients was 0.733. Furthermore, a nomogram was established and could be used clinically to stratify patients to predict prognosis. Patients defined as high-risk by this signature showed a lower proportion of eosinophils than the low-risk group. Patients with low risk were more sensitive to anti-CTLA4 immunotherapy. Additionally, HSPA1A and FAM50B were two SF regulating the OS-related AS. Downregulation of HSPA1A and FAM50B inhibited the proliferation and migration of Caco2 cells.

**Conclusion:** We constructed an ideal prognostic signature reflecting the genomic instability and OS of CRC patients. HSPA1A and FAM50B were verified as two important SF regulating the OS-related AS.

## Introduction

Colorectal cancer (CRC) is the second most common cancer diagnosed in women and the third most common in men (Dekker et al., 2019). It is the second leading cause of cancer deaths (Keum and Giovannucci, 2019). In 2020, 1,148,515 new cases of CRC were diagnosed, and about 576,858 individuals died from malignancy (Sung et al., 2021). CRC is a multifactorial disease characterized by molecular and clinical heterogeneity (Nguyen et al., 2020). Therefore, it is urgently necessary to explore novel biomarkers for improving the clinical outcome of CRC patients.

Alternative splicing (AS) is one of the essential post-transcriptional regulatory mechanisms and contributes to enriching the protein diversity from a limited number of genes (Baralle and Giudice, 2017; Sciarrillo et al., 2020). Increasing evidence has suggested that aberrant alternative splicing (AS) events regulate cell proliferation, invasion, apoptosis, angiogenesis, and drug resistance, resulting in the progression of CRC (Chen et al., 2021). Alternatively spliced CD44 variants have been identified to promote intestinal tumorigenesis induced by the activation of Wnt signaling (Guo and Frenette, 2014). In chemoradiation-resistant colon cancer cells, exon skipping is significantly increased (Xiong et al., 2016). Alternative splicing isoforms of vascular endothelial growth factor A (VEGFA), UDP glucuronosyltransferase family 1 member A complex locus (UGT1A), pregnane X receptor (PXR), and KRAS are potential therapeutic targets for CRC (Audet-Delage et al., 2017; Canavese et al., 2017; Eilertsen et al., 2019).

Genomic instability, an important prognostic factor of cancer, has been reported to be a hallmark of cancer (Duijf et al., 2019). The instability is multifaceted at several different levels, ranging from simple deoxyribonucleic acid sequence changes to chromosomal aberrations. The molecular mechanisms underlying genomic instability implicate numerous levels of gene regulation, such as transcriptional and post-transcriptional regulation (Chen et al., 2022). Studies indicated that 92–94% of human genes undergo AS (Wang et al., 2008). In addition, multiple AS events have been identified to be associated with genomic instability (Liu et al., 2020; Sebastian et al., 2020a). The dysregulated AS disturbs genome integrity resulting in tumorigenesis (Öther-Gee Pohl and Myant, 2022). However, whether the AS events could reflect the genomic instability and overall survival (OS) of CRC is currently unknown.

In this study, we compared the differential AS events between genomic stable and unstable patients. We developed a new prognostic model combining AS profiles and somatic mutation profiles in CRC tumor tissues. In addition, we explored the related splicing factors and infiltrating immune cells in this prognostic model. Our studies identified the potential molecular signature as genomic instability-associated CRC biomarkers, which may be helpful to assess the clinical outcomes of CRC patients accurately.

## Materials and methods

### Data collection

Clinical information, RNA-seq data, and somatic mutation data of CRC cohorts were obtained from The Cancer Genome Atlas (TCGA) database (https://tcga-data.nci.nih.gov/). AS events from CRC patient samples (*n* = 442) were collected from TCGAspliceSeq database (http://bioinformatics.mdanderson.org). The SpliceSeq tool was used to analyze AS profiles and assess the splicing patterns of mRNA in CRC. The Percent Splicing index (PSI), ranging from zero to one, was used to quantify AS events. AS events with PSI values >75% were selected. The AS events were visualized by using the R package: UpSetR (v1.4.0).

### Screening of the genomic instability-related alternative splicing events

**FIGURE 1:**
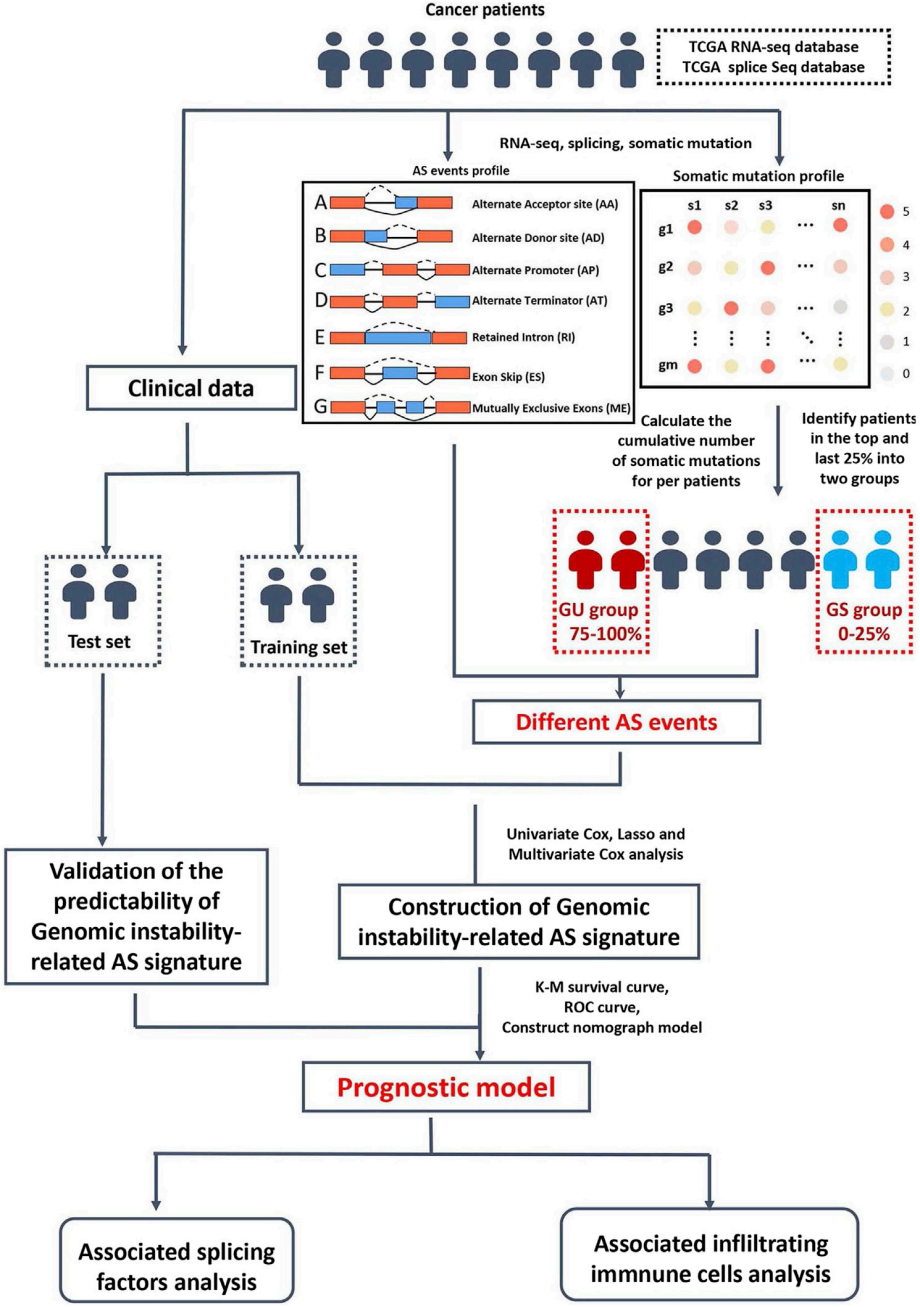
Study flowchart.

A computational framework was performed to analyze genomic instability-related AS events by combining the PSI values of the AS events and somatic mutation profiles (Figure 1). The somatic mutation quantity of each patient in TCGA database were calculated with “varscan”. We then ranked each patient’s somatic mutations number in descending order, then defined the top 25% (*n* = 98) and the last 25% (n = 97) of the patients as genomic unstable (GU) group and genomic stable (GS) group, respectively. To filter differentially expressed AS events which were defined as genomic instability-related AS events, the significance analysis of microarrays (SAM) method was used to compare the PSI values between these two groups (*p* < 0.05).

### Identification of survival-associated alternative splicing events and the prognostic signature construction

Univariate Cox analysis was performed to determine the survival-associated genomic instability-related AS events. To remove high correlations among the seven AS events, we used the LASSO (Least absolute shrinkage and selection operator) regression. Multivariate Cox regression analyses were performed to estimate the independent predictor function of each AS event. Finally, we calculate the risk score using the following formula: $\text{Risk}\;\text{score}=\overset{n}{\underset{i}{\sum }}PSIi\times \beta i$ , where β denotes the regression coefficient of each event. Then the median of risk score was defined as a cut-off value distinguishing the high-and low-risk groups. The LASSO regression was performed with “glmnet” and “survival” R package. The multivariate Cox regression analyses were performed with “survival” R package.

### Validation of the prognostic signature

To provide additional support to our findings, the 433 CRC patients were randomly divided into a training set (*n* = 217) and a test set (*n* = 216) using the R package “caret” (Table1 summarizes the clinicopathological characteristics of the CRC patients). First, each set of CRC patients was divided into high-risk and low-risk groups using the same formula to compute the risk score. Kaplan–Meier survival curve and Log-Rank test were used to compare overall survival between the high-risk and low-risk groups in the training set. The survival receiver operating characteristic (ROC) package (R 4.0.3) was then used to assess the ability of the prediction model. Univariate and Multivariate Cox regression were conducted to calculate the high-risk score’s hazard ratio (HR). Subsequently, we validated the prognostic signature in the test set and the entire TCGA dataset (*n* = 433). Kaplan–Meier, Cox, and ROC analyses were carried out as described above.

**TABLE 1:** Clinicopathological information of the patients with CRC in TCGA.

Covariates	Type	Total (*n* = 433)	Test set (*n* = 216)	Training set (*n* = 217)	*p*-value
Age (%)	≤65	178 (41.11%)	88 (40.74%)	90 (41.47%)	0.9541
	>65	255 (58.89%)	128 (59.26%)	127 (58.53%)	
Sex (%)	Female	204 (47.11%)	101 (46.76%)	103 (47.47%)	0.9594
	Male	229 (52.89%)	115 (53.24%)	114 (52.53%)	
Tumor stage (%)	Stage I-II	244 (56.35%)	123 (56.94%)	121 (55.76%)	0.8317
	Stage III-IV	178 (41.11%)	87 (40.28%)	91 (41.94%)	
	Unknow	11 (2.54%)	6 (2.78%)	5 (2.3%)	
T stage (%)	T1-2	86 (19.86%)	44 (20.37%)	42 (19.35%)	0.8662
	T3-4	346 (79.91%)	171 (79.17%)	175 (80.65%)	
	Unknow	1 (0.23%)	1 (0.46%)	0 (0%)	
M stage (%)	M0	318 (73.44%)	163 (75.46%)	155 (71.43%)	0.6089
	M1	60 (13.86%)	28 (12.96%)	32 (14.75%)	
	Unknow	55 (12.7%)	25 (11.57%)	30 (13.82%)	
N stage (%)	N0	259 (59.82%)	129 (59.72%)	130 (59.91%)	1
	N1-2	174 (40.18%)	87 (40.28%)	87 (40.09%)	

### Immune cell analysis

We used the CIBERSORT algorithm (http://cibersort.stanford.edu/) to estimate the 22 kinds of infiltrating immune cells in CRC tissue. A total of 163 cases were included for further analysis with *p*-values <0.05. The median risk scores classified these cases into high-risk (*n* = 81) and low-risk (*n* = 82) groups. The R package “vioplot” was used to draw the differential immune cells types between these two groups. The survival curve was generated using the R package “survival”. The P- value is calculated based on the log-rank. Immunophenoscore (IPS) data were obtained from The Cancer Immunome Atlas (TCIA) database (https://tcia.at/), predicting the response to cytotoxic T lymphocyte antigen 4 (CTLA4) and programmed cell death protein 1 (PD-1) blockers (*n* = 433). According to the median risk scores classified these cases into high-risk (*n* = 216) and low-risk (*n* = 217) groups. The differential effective immunotherapy responses between high-risk and low-risk groups were analyzed by the chi-square test and visualized by R package “vioplot”.

### Correlation network of survival associated alternative splicing events and splicing factors construction

A total of 404 splicing factor genes were identified in a previous study(Seiler et al., 2018). The mRNA profile data of the splicing factors (SF) in CRC were obtained from the TCGA database. Correlations between the splicing factor expression and prognosis-related AS events were visualized and analyzed by Cytoscape 3.7.2. In Univariate Cox regression, a *p*-value of <0.05 and correlation coefficient >0.1 were identified as statistically significant.

### Cell culture and transfection

Caco2 cells were purchased from ATCC (Manassas, VA, United States) and cultured with a DMEM-High glucose medium (HyClone, Logan, UT, United States) supplied with 10% fetal bovine serum (FBS, Lonsera, UY). and 50 U/ml Penicillin-G, 50 µg Streptomycin (Thermo Scientific, Cambridge, MA, United States). Cell cultures were maintained at 37°C in a humidified atmosphere of 5% CO2.

The small interfering RNA of HSPA1A and FAM50B and negative control were purchased from TsingkeBiotechnology (Beijing, CHN). For the siRNA experiments, cells were seeded in 6-well plates at a density of 5 x 10^4 cells/well and medium was replaced with serum-free medium once confluence reached ∼80%. Subsequently, 160 pmol siRNA (80 pmol of each siRNA when two siRNAs were co-transfected) was mixed with 200 μl serum-free medium containing 8 μl Lipofectamine RNAiMAX Reagent (Invitrogen, United States) and added to the cells. In the following experiments, the cells were divided into four groups: control siRNA (NCi) group, HSPA1A siRNA (HSPA1Ai) group, FAM50B siRNA (FAM50Bi) group, HSPA1A siRNA + FAM50B siRNA (HSPA1Ai + FAM50Bi) group. The oligonucleotide sequences were as follows: siRNA-HSPA1A sense strand, 5′- GCC​AUG​ACG​AAA​GAC​AAC​ATT-3′ and antisense strand, 5′-UGU​UGU​CUU​UCG​UCA​UGG​CTT -3’; siRNA-FAM50B sense strand, 5′-GCU​GGU​ACG​AGA​ACA​ATT -3′ and antisense strand, 5′-UUG​UUC​UUC​UCG​UAC​CAG​CTT -3’. The primer sequence used were listed in Table2.

**TABLE 2:** Primers used in this study.

Name	Primer (5′–3′)
HSPA1A	Forward: CAT​CAT​CAG​CGG​ACT​GTA​CCA
	Reverse: TGC​AAA​CAC​AGG​AAA​TTG​AGA​AC
FAM50B	Forward: AAG​AGG​TTC​TCG​GCG​CAT​TAC
	Reverse: CGG​GCC​TTC​ATG​TCG​TTC​A
36B4	Forward: CAG​CAA​GTG​GGA​AGG​TGT​AAT​CC
	Reverse: CCC​ATT​CTA​TCA​TCA​ACG​GGT​ACA​A

### RNA isolation and real-time qPCR

Total RNA of siRNA infected cells was extracted using RNAiso Plus (Takara), according to the manufacture’s protocols. Subsequently, the isolated RNA was reverse transcribed into cDNA with PrimeScriptTM Reagent Kit (Takara). The reaction mixture for qPCR containing SYBR (BAOGUAGN, China) was prepared according to the manufacture’s protocols. RT-PCR was performed in a PCR system with HSPA1A, FAM50B, and 36B4 primers. Relative gene expression was calculated using the 2^−△△CT^ method, using 36B4 mRNA expression as reference gene. Each sample was analyzed at least three times.

### Cell proliferation experiments

Caco2 cells were seeded in 96-well plates (Corning, NY, United States) (3*10^3 cells/well) and transfected with siRNA for 24 h. Then these cells were cultured for 4 days, and the cell proliferation was measured using a CCK8 reagent (Beyotime, Beijing, CHN).

### Wound healing assay

The Caco2 cells were seeded in 6-well plates at a density of 5 × 10^4 cells/well. After 24 h of siRNA transfection, wounds were made in center of the well using a sterile 10ul pipette tip. Images of five randomly-selected scratched fields were captured on an inverted light microscope (ZEISS, Oberkochen, BE, GER) at 0 and 24 h. Magnification, X200. Wound areas were measured by Image J, and calculated the wound healing percentage.

### Transwell assay

Cells were added to the upper chambers of the Transwells (Cornning, NY, United States) at a density of 5 × 10^5 cells/well and transfected with siRNA. After 24 h, medium in the lower chamber of a transwell was exchanged for complete medium and medium in the upper chamber of a transwell was exchange for serum-free medium. After the cells were cultured in transwell for 24 h, cells on the lower layer of the membrane were fixed by 4% paraformaldehyde (Sangon, Shanghai, CHN) for 10 min, then stained using Crystal Violet Staining Solution (Beyotime) for 30min. The cells number was counted by using an inverted microscope (ZEISS) and five fields were randomly selected to count the cells. Magnification, ×200.

### Statistical analysis

Statistical analyses were performed using the statistical package GraphPad Prism, version 8.0 (California, United States). All results are expressed as mean ± SEM. Student’s t-tests were used to compare results between two groups, and One-way ANOVA was used to compare differences among multiple groups. Results were considered statistically significant at *, #, *p* < 0.05.

## Results

### Screening of genomic instability-related alternative splicing events in colorectal cancer patients

**FIGURE 2:**
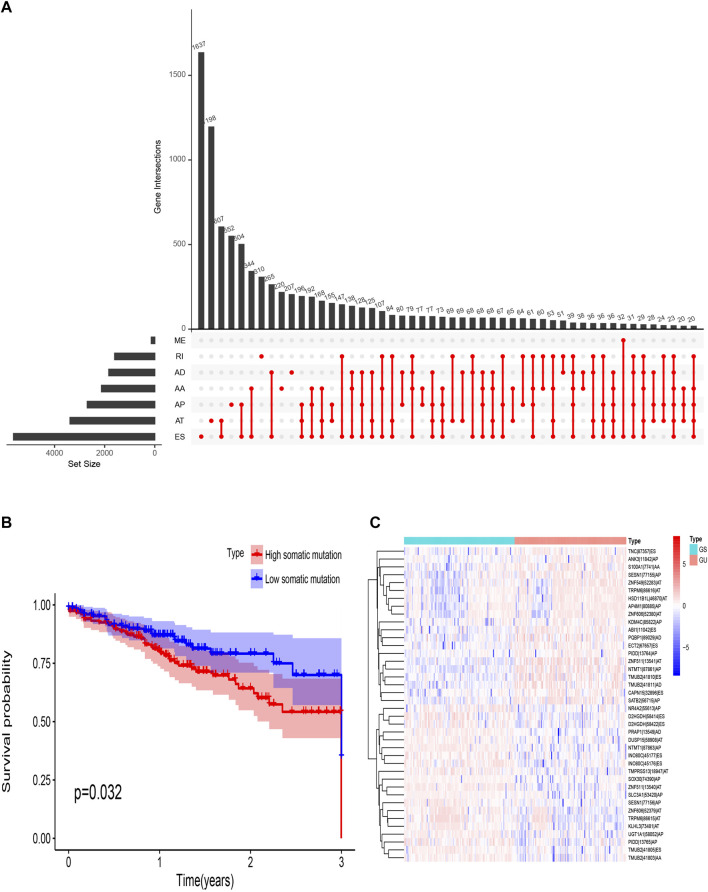
Overview of AS events in TCGA CRC cohort. **(A)** Upset plot for all AS events. AS, alternative splicing; RI, retained intron; ME, mutually exclusive exons; ES, exon skipping; AT, alternative terminator; AP, alternative promoter; AD, alternative donor site; AA, alternative acceptor site. **(B)** Survival probability of different somatic mutation group. **(C)** Heat map of genomic instability-related AS events. TCGA, The Cancer Genome Atlas; CRC, Colorectal cancer.

There are seven types of AS events: Mutually Exclusive Exons (ME), Retained Intron (RI), Alternate Donor site (AD), Alternate Acceptor site (AA), Alternate Terminator (AT), Alternate Promoter (AP), and Exon Skip (ES). In this study, 442 CRC patients were included. Seventy-four ME events in 74 genes, 1655 RI in 1184 genes, 1691 AD events in 1316 genes, 2006 AA events in 1576 genes, 3973 AP events in 2330 genes, 4710 AT events in 2783 genes, and 8416 ES events in 4436 genes. In TCGA-CRC, ES events were the most frequent type of spliced signatures, followed by AT and AP events, and ME was the least frequent (Figure 2A). Next, we divided TCGA CRC patients into high-somatic mutation and low- somatic mutation groups according to the median of the somatic mutation counts of each patient. As shown in Figure 2B, the three-year OS was significantly lower in patients with higher level of somatic mutation (*p* = 0.032), demonstrating a key role of genomic instability in the OS of CRC patients. Furthermore, we defined patients with the top 25% (*n* = 98) and the last 25% (*n* = 97) of somatic mutation counts as genomic unstable (GU) group and genomic stable (GS) group, respectively. The mean somatic mutation numbers of GS and GU groups were 88 and 2058, respectively. A heat map of the top 40 different AS events is showed in Figure 2C.

### Construct a genomic instability-related alternative splicing signature for overall survival in the training set

**FIGURE 3:**
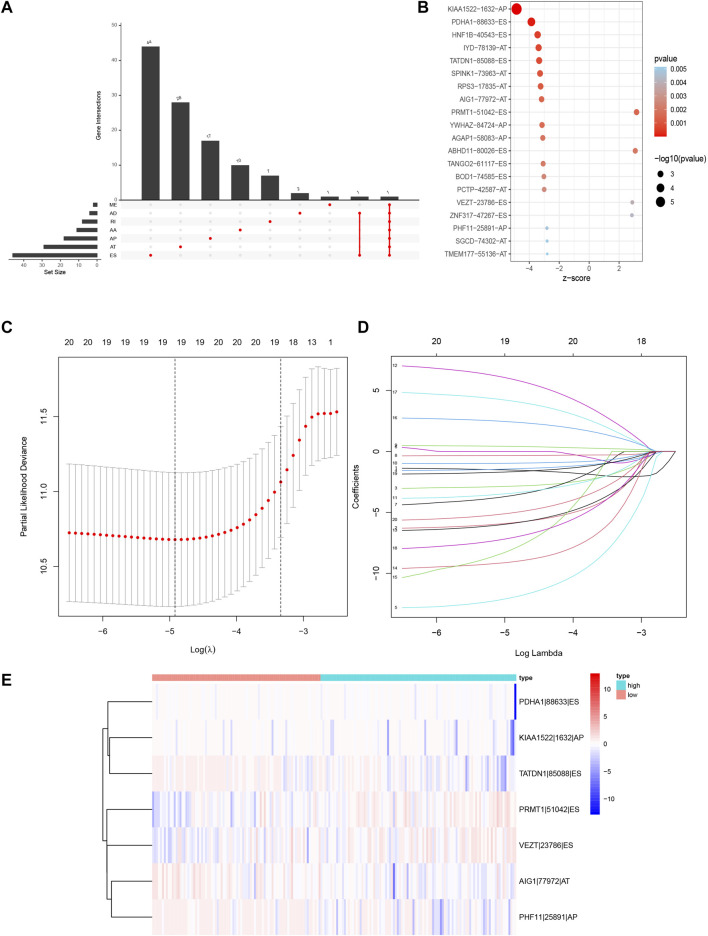
Prognosis-related AS events in this study. **(A)** Upset plot for survival-related genes. **(B)** The Bubble plots of survival-associated AS events in CRC. **(C,D)** Optimal survival-related AS events selection in the LASSO regression model. **(E)** Heat map of the seven optimal survival-related AS events. LASSO, least absolute shrinkage and selection operator.

We screened the AS events related to the OS of CRC patients. The results showed that 114 AS events were significantly associated with OS in CRC patients (Figures 3A,B). Then Lasso Cox regression analysis was used to further select the AS events related to the OS and prognosis of CRC patients (Figures 3C,D). Then, the risk score was calculated for each AS event (Table3). Seven AS events: PDHA1-88633-ES, KIAA1522-1632-AP, TATDN1-85088-ES, PRMT1-51042-ES, VEZT-23786-ES, AIG1-77972-AT, and PHF11-25891-AP were identified as independent risk factors for OS in CRC using multivariate Cox regression. Moreover, two AS events (PRMT1-51042-ES, VEZT-23786-ES) had positive coefficients suggesting that high expression of these two AS events were associated with poorer prognosis as risk factors. In contrast, the remaining AS events (PDHA1-88633-ES, KIAA1522-1632-AP, TATDN1-85088-ES, AIG1-77972-AT, and PHF11-25891-AP) had negative coefficients suggesting that their upregulated expression were associated with better survival as protective factors. Next, a genomic instability-related AS events prognostic signature was established by quantifying the PSI of the seven AS events and their coefficients from the multivariate Cox regression analysis. Risk Score = (−6.779 × PSI of PDHA1-88633-ES) + (−2.957 × PSI of KIAA1522-1632-AP) + (−36.891 × PSI of TATDN1-85088-ES) + (3.553 × PSI of PRMT1-51042-ES) + (5.388 × PSI of VEZT-23786-ES) + (−4.116 × PSI of AIG1-77972-AT) + (−9.906 × PSI of PHF11-25891-AP). The heatmap of the seven AS events is shown in Figure 3E.

**TABLE 3:** Details of the selected AS events based on multivariate Cox analysis.

AS event	Coefficient	HR	95% CI	*p*-value
KIAA1522-1632-AP	−2.957	0.052	0.003–1.048	0.054
PDHA1-88633-ES	−6.779	0.001	1.77E-05–0.073	0.001
TATDN1-85088-ES	−36.891	9.52E-17	2.58E-27–3.51E-06	0.003
AIG1-77972-AT	−4.116	0.016	0.0004–0.670	0.030
PRMT1-51042-ES	3.553	34.913	3.040–400.990	0.004
VEZT-23786-ES	5.388	218.699	9.810–4875.360	0.001
PHF11-25891-AP	−9.906	4.99E-05	3.24E-08–0.077	0.008

### The alternative splicing signature was associated with genomic instability in colorectal cancer patients

**FIGURE 4:**
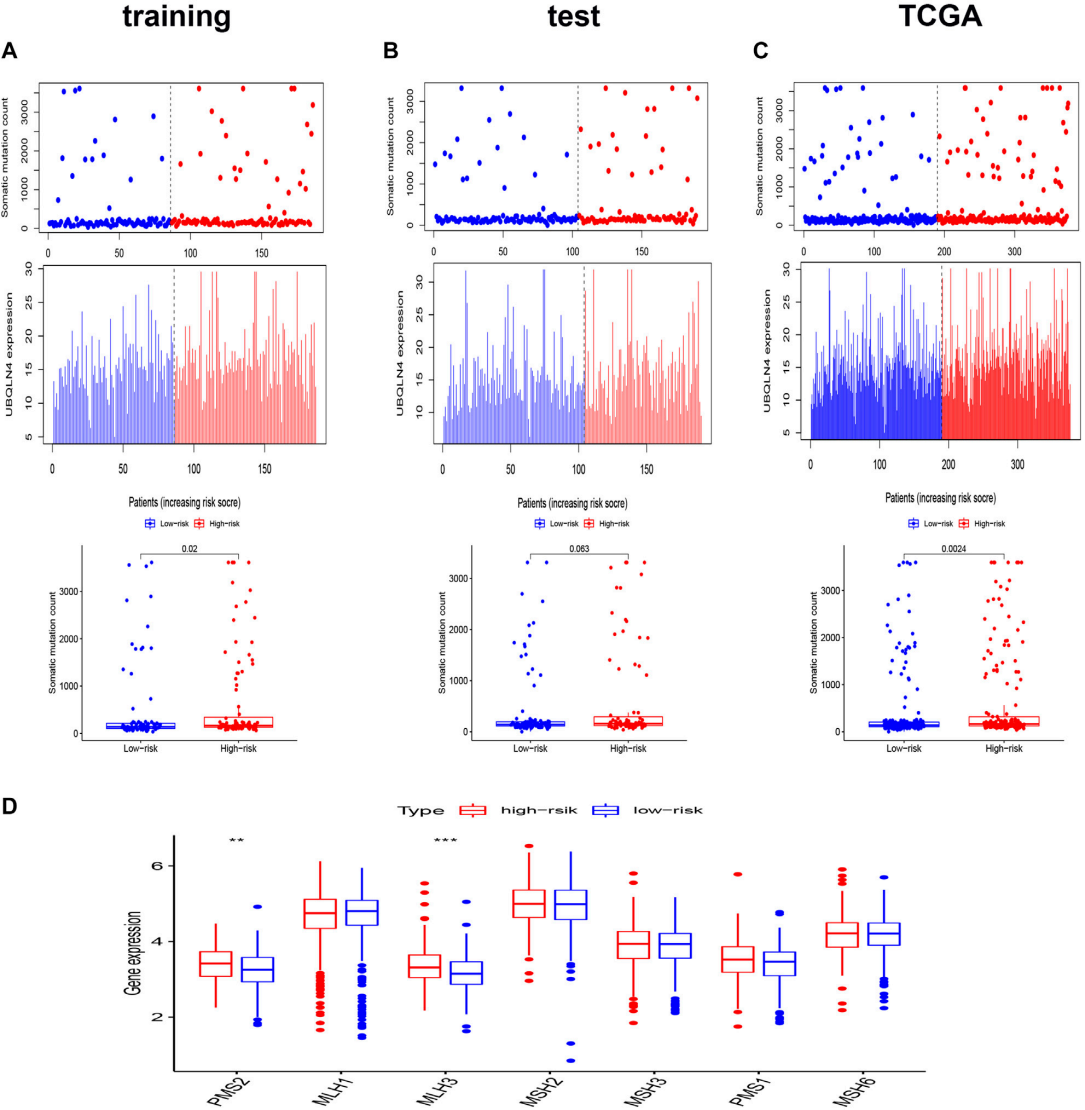
Relationship between the genomic instability-related AS signature and somatic mutation patterns of CRC patients. **(A–C)** The distribution of somatic mutation count and UBQLN4 expression of the training set **(A)**, the test set **(B)**, and the entire TCGA set **(C)**. **(D)** Boxplots comparing the seven DNA mismatch repair genes expression between high- and low-risk groups. *p < 0.05 high-risk group vs*.* low-risk group. Statistical analysis was performed using the Mann-Whitney U test.

To verify whether the AS signature was associated with the somatic mutation pattern, we compared the expression of UBQLN4, a biomarker for driving genomic instability (Jachimowicz et al., 2019), between the two different risk groups in the three sets. As shown in Figures 4A–C, the expression of UBQLN4 and somatic mutation count was higher in the high-risk groups than in the low-risk group, in which the *p* values were 0.02 in the training set, 0.063 in the test set, and 0.0024 in the entire TCGA set. These results implied that the AS signature score was associated with genomic instability. Furthermore, DNA somatic mismatch repair (MMR) genes associated with genomic instability (Baross-Francis et al., 1998). We then analyzed the genomic alterations of seven DNA mismatch repair genes (MLH1, MLH3, MSH2, MSH3, MSH6, PMS1, PMS2) in the two different risk score groups. As shown in Figure 4D, the gene expression of PMS2 and MLH3 were significantly higher in the high-risk group. These results reconfirm that our prognostic signature was associated with genomic instability.

### Predictability evaluation of genomic instability-related alternative splicing signature in colorectal cancer patients

**FIGURE 5:**
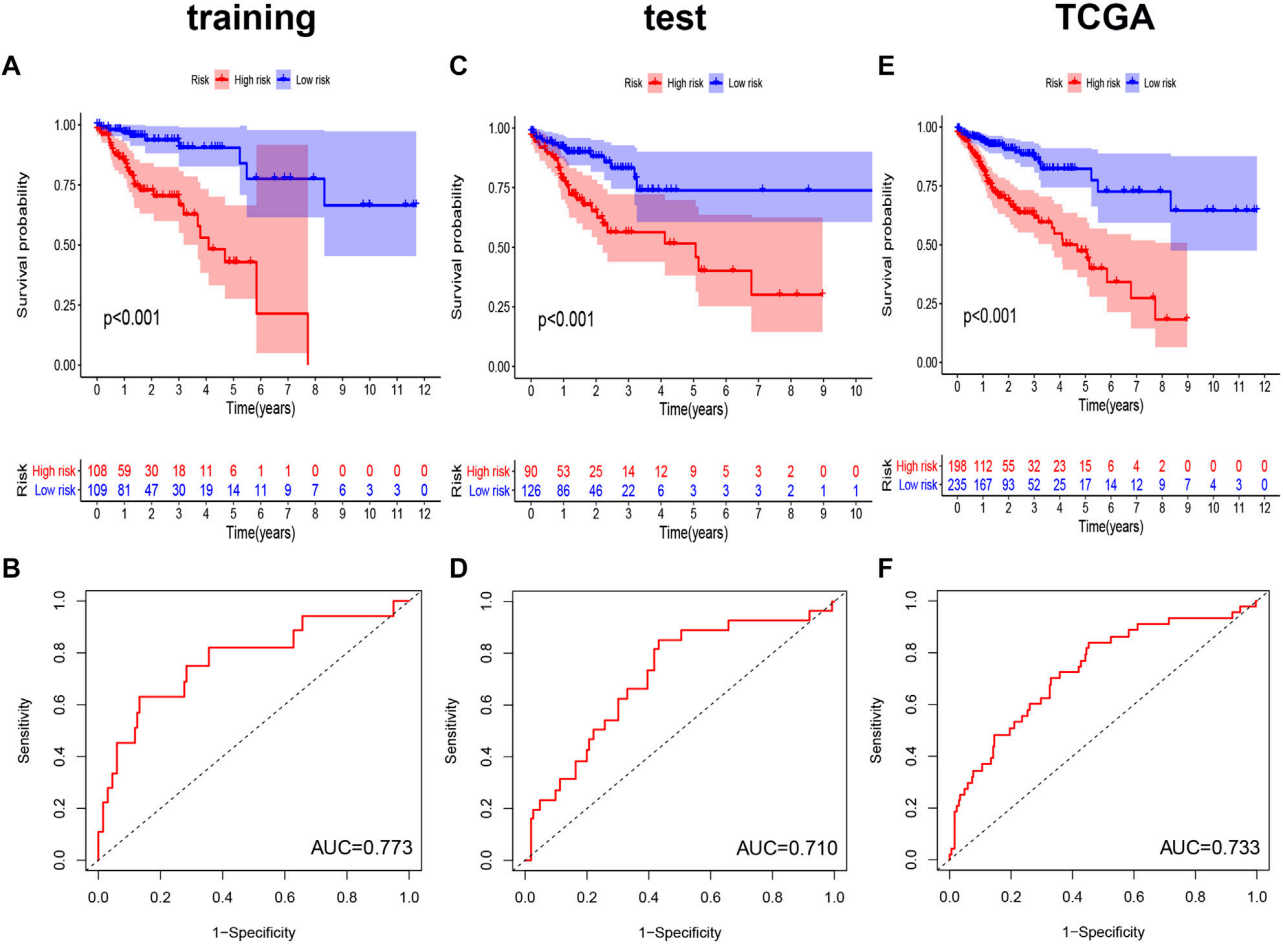
Kaplan-Meier curves and ROC curves of the prognostic AS models. **(A,C,E)** Kaplan-Meier plots of the genomic instability-related AS signature in the training set **(A)**, the test set **(C)**, and the entire TCGA set **(E)**. **(B,D,F)** The ROC curves for overall survival of the genomic instability-related AS signature in three datasets, respectively. ROC, Receiver operating characteristic.

We performed Kaplan-Meier analysis and constructed a ROC curve to verify the prognostic efficiency of the AS signature in the training set (Figures 5A,B). The risk score distribution curves showed that higher risk score patients in CRC had a shorter survival time. Kaplan-Meier survival curve analysis verified that patients with higher risk scores had poorer OS, *p* < 0.001. Moreover, the AUC value of the predictive accuracy of the model was 0.773. To further validate the prognostic significance of the genomic instability AS signature, we calculated the genomic instability AS signature scores of the test set and the entire TCGA set and constructed the respective ROC curves. In the test set, the survival of the low-risk group was significantly longer than that of the high-risk group, with an AUC value of 0.710. (Figures 5C,D). Similar results were also obtained in the entire TCGA set, where the AUC of the ROC curves was 0.733 (Figures 5E,F). These results suggested that genomic instability-related AS signature had a good survival prediction efficacy.

### The genomic instability-related alternative splicing signature was independent of other clinical factors

**FIGURE 6:**
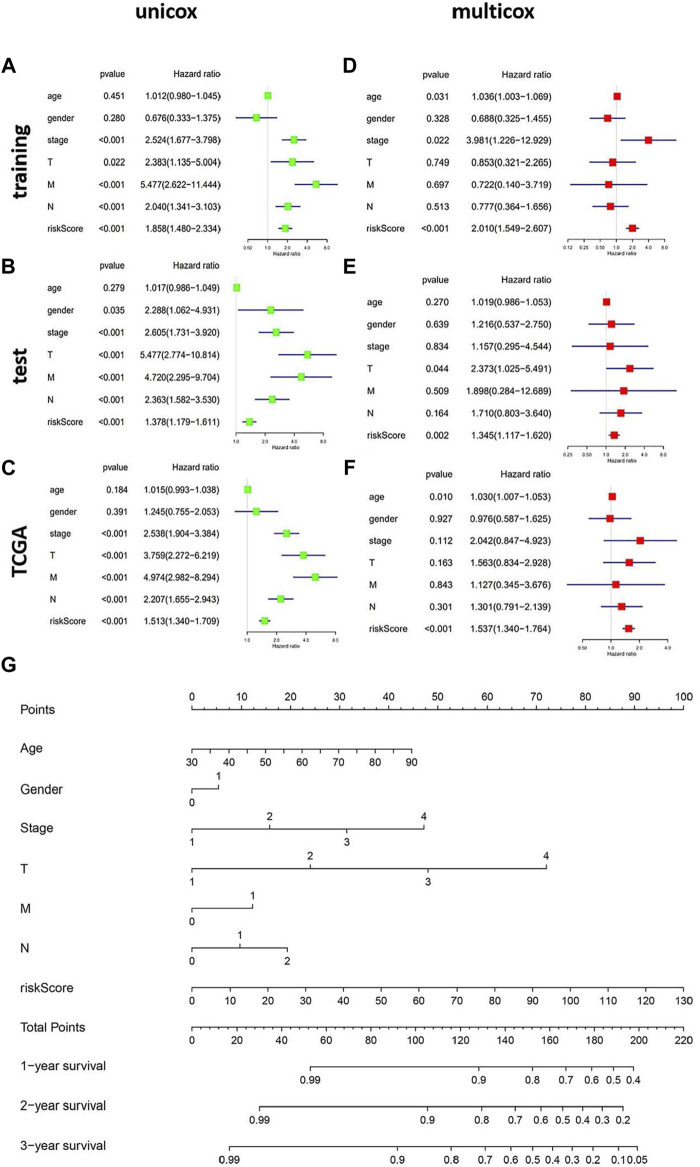
The independent prognostic analysis of the genomic instability-related AS signature. Construction of the nomograph model in patients with CRC. (**A–C**) Forest plots of univariate cox regression in the training set, test set, and the entire TCGA set. (**D–F**) Forest plots of multivariate cox regression in the three datasets. (**G**) The nomograph model predicting 1-, 2-, and 3-year survival in patients with CRC based on age, sex, TMN stage, and risk score.

To evaluate whether the genomic instability-related AS signature could act as an independent prognostic factor of clinicopathological features, univariate and multivariate Cox regression analyses were performed, adjusting for age, sex, and pathologic stage in the three data sets (training, test, and TCGA data set) (Figures 6A–C). The univariate Cox regression results showed that with the OS, genomic instability AS signature and tumor stage were significantly correlated in these three data sets (*p* < 0.001). The prognostic significance of each data set was also retained in multivariate Cox regression analyses. For other clinical features, only sex had a significant correlation with the genomic instability-related AS signature in the test set using univariate Cox regression analyses (*p* = 0.035) (Figures 6D–F). Moreover, to make this model more practicable in the clinic, we constructed a nomograph model based on the risk score, tumor stage, age, and sex to predict the 1-, 2-, and 3- year survival of patients with CRC (Figure 6G).

### Tumor-infiltrating immune cells were associated with the prognostic alternative splicing signature

**FIGURE 7:**
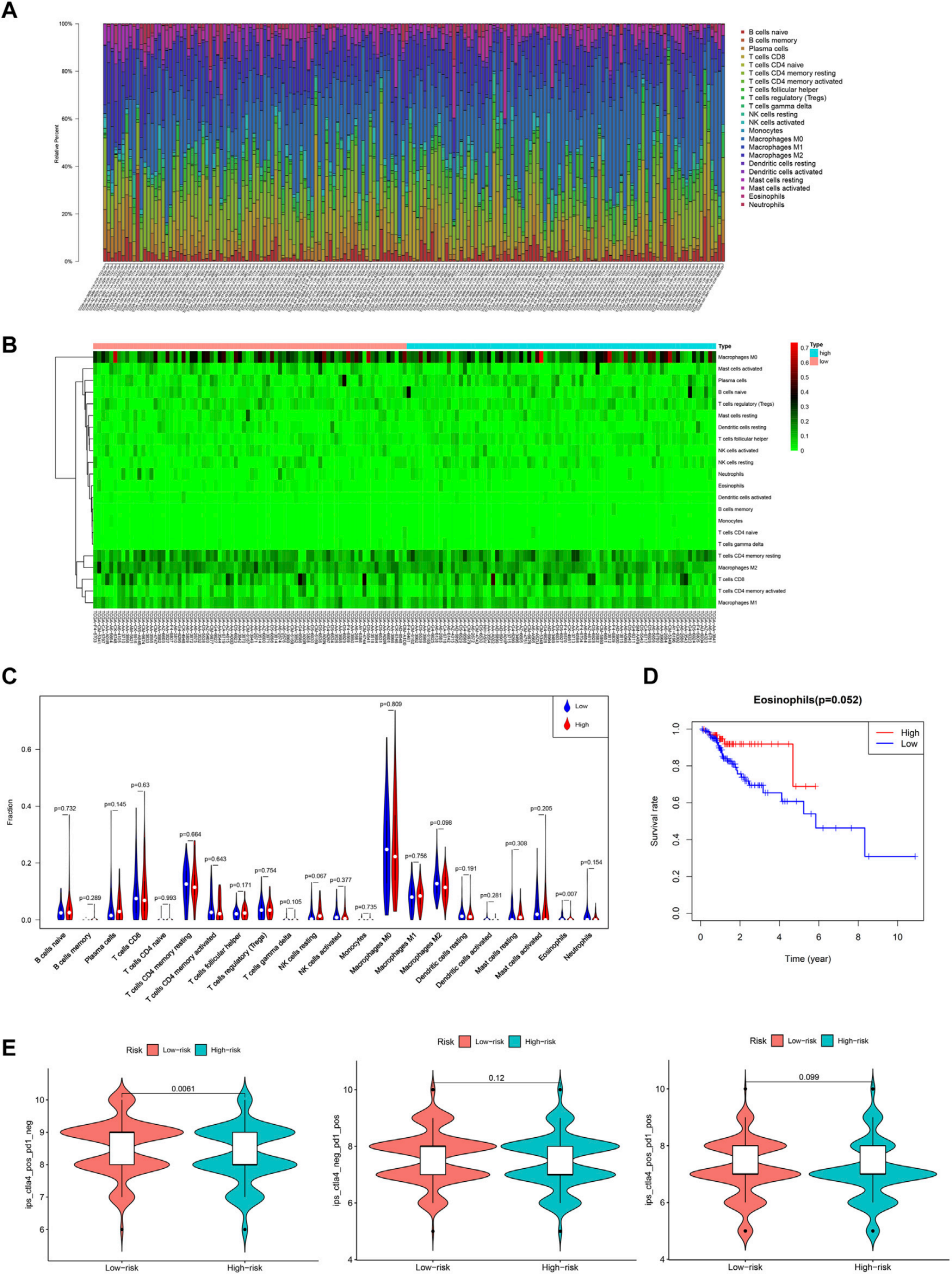
Overview of the infiltrating immune cells in CRC. **(A)** Bar plot showing the proportion of the 22 types of immune cells. **(B)** Heat map of the immune cells proportion between the high- and low-risk groups. **(C)** Comparison of each immune cell type in the two risk groups. **(D)** Kaplan-Meier estimates of overall survival of patients with low or high eosinophils expression. **(E)** Violin plots of the IPS in two risk groups. IPS, immunophenoscore.

To investigate the relationship between tumor-infiltrating immune cells and the prognostic AS signature, we used CIBERSORT to identify tumor-infiltrating immune cells in 163 CRC patients. Detailed tumor-infiltrating immune cell information on each patient was illustrated in Figure 7A **.** Furthermore, according to the risk score, we divided these patients into high-risk and low-risk groups (Figure 7B). As shown in Figure 7C, compared with the low-risk group, the high-risk group had a lower proportion of eosinophils (*p* = 0.007). Additionally, we explored the relationship between the proportion of eosinophils and clinicopathological information of CRC patients. Next, we used a ROC curve to verify the prognostic efficiency of eosinophils expression, and the results showed that higher eosinophils expression had higher OS rates (*p* = 0.052) (Figure 7D). We also explored the association between immunotherapy efficiency between the high-risk and low-risk groups. As shown in Figure 7E, patients with low risk were more sensitive to targeting CTLA4 treatment. For PD-1 alone or in combination with CTLA4 treatment, there were no significant differences between the two risk groups. Therefore, these data implied that our prognostic AS signature might facilitate immunotherapy results prediction.

### Exploring the regulatory network of regulating the prognostic alternative splicing signature

**FIGURE 8:**
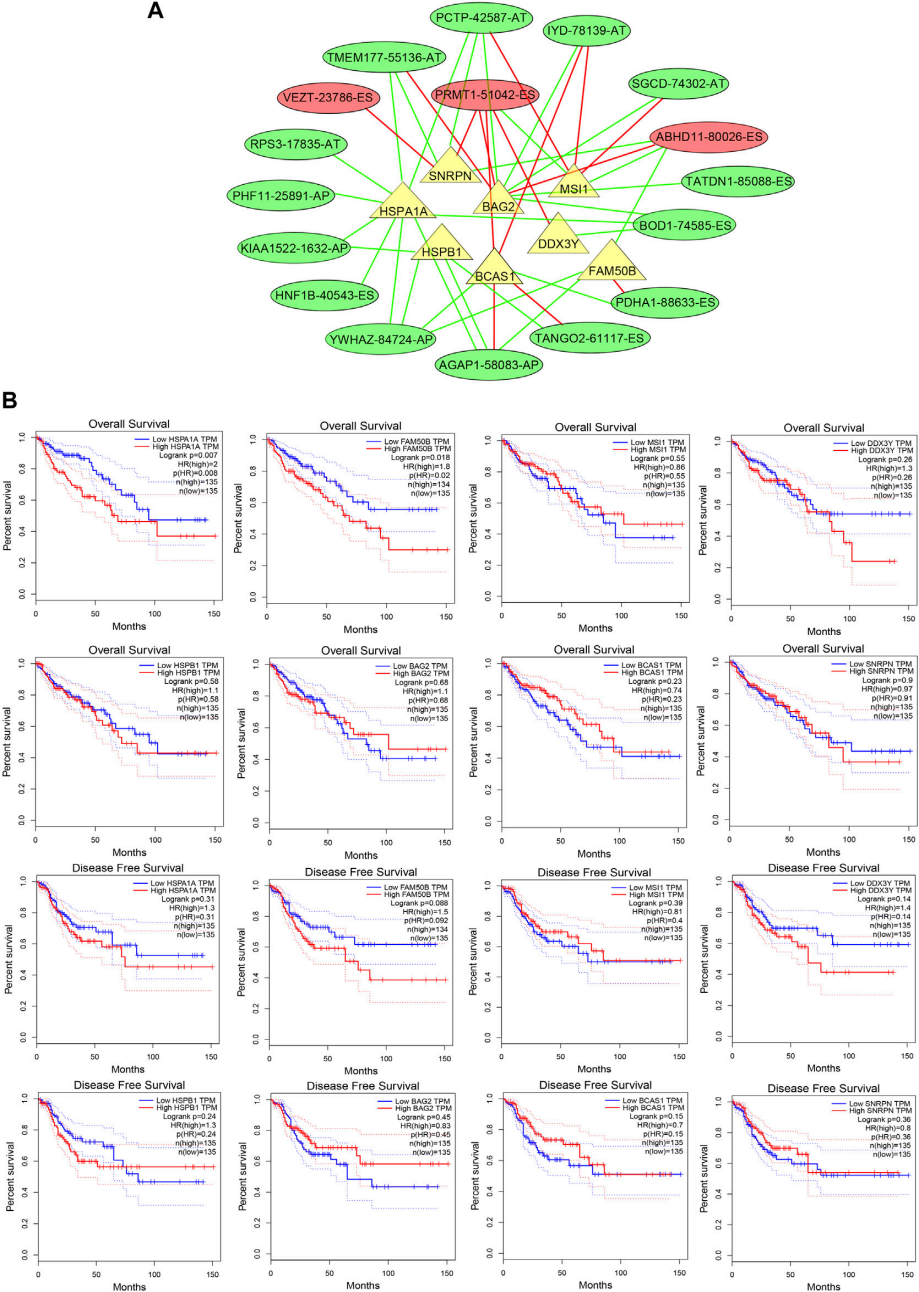
The splicing factors are associated with prognostic AS signature. **(A)** Splicing correlation network in CRC. The triangles represent the survival-related SF. The red and green ovals represent SREs that increase and decrease risk, respectively. Red and green lines represent the positive and negative correlations of connected triangles, respectively. SRE, alternative splicing events; SF, splicing factor. **(B)** ROC analysis of overall survival and disease-free survival for the AS signature-related splicing factors in patients with CRC. SF, splicing factor.

Due to the unavailability of inhibiting AS specifically, we explored the regulatory network for regulating the prognostic AS signature and tried to find a target regulating OS-related AS. We constructed a splicing-regulatory network to further determine whether the prognostic AS events were regulated by specific splicing factors in CRC. As shown in Figure 8A, eight splicing factors (SNRPN, HSPA1A, HSPB1, BAG2, BCAS1, DDX3Y, MSI1, and FAM50B) were significantly correlated with survival-associated AS events, and more than half of the survival-related AS events were regulated by more than one splicing factor. Furthermore, we assessed the function of these splicing factors in the prognosis of CRC patients (Figure 8B). The result showed that patients with lower HSPA1A expression levels and FAM50B expression levels had longer OS rates; the *p-*values were 0.008 and 0.02, respectively. Although the *p*-value approached insignificance, lower FAM50B expression also showed longer disease-free survival rates (*p* = 0.092).

### Validation the effect of HSPA1A and FAM50B in Caco2 cells proliferation and migration

**FIGURE 9:**
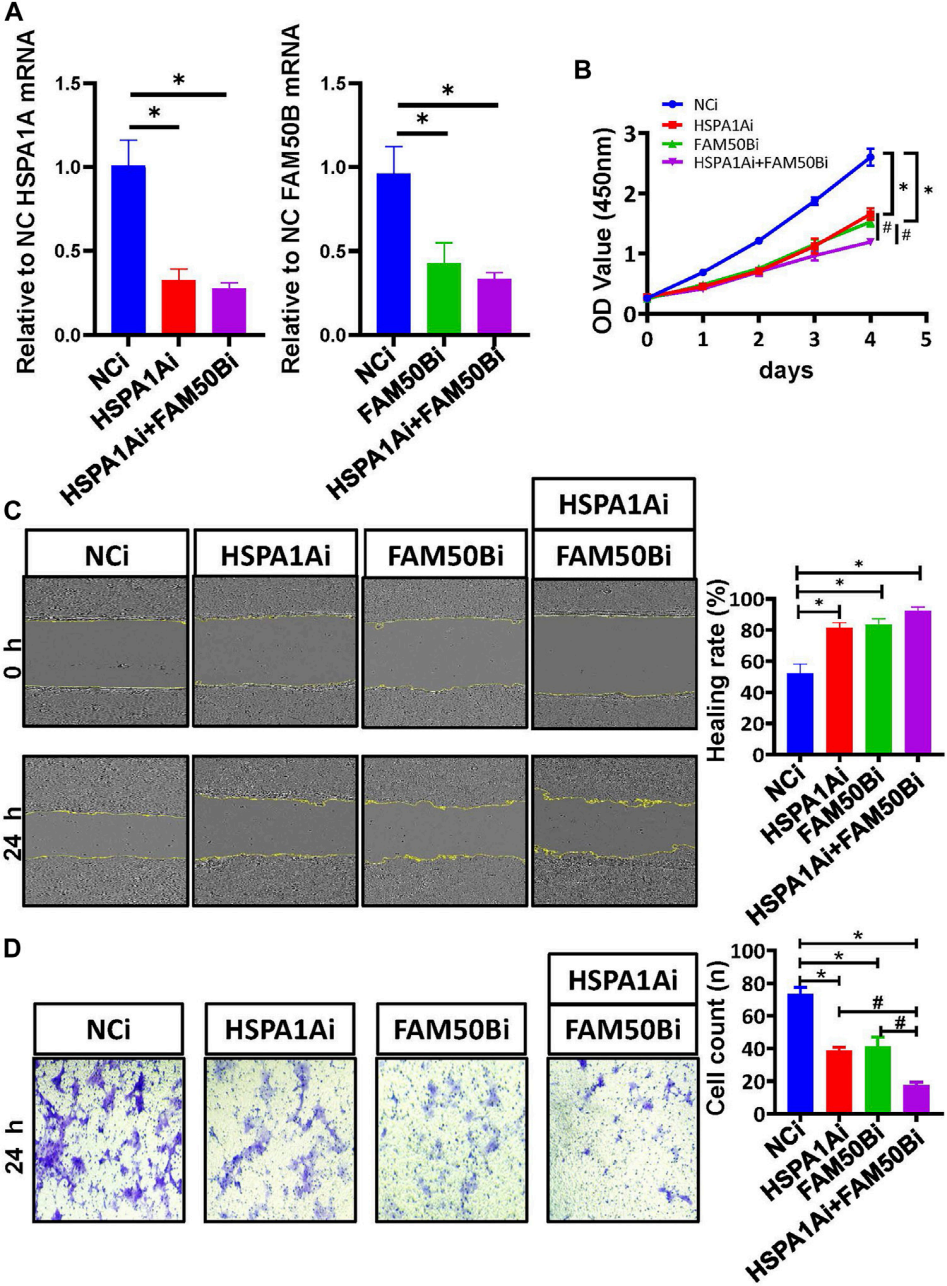
Downregulation of HSPA1A and FAM50B inhibits the proliferation and migration of Caco2 cells. Caco2 cells were transfected with control siRNA, HSPA1A siRNA, and FAM50B siRNA, respectively, or co-transfected with HSPA1A siRNA and FAM50B siRNA. **(A)** The mRNA levels of HSPA1A and FAM50B in Caco2 cells after transfection. **(B)** Cell proliferation were assessed by CCK8 assays (n ≥ 4). **(C and D)** The cell migration was detected by scratch **(C)** and transwell assays **(D)** (*n* = 3). * *p* < 0.05 vs. NCi group. #*p* < 0.05 vs. HSPA1Ai + FAM50Bi. Data are mean ± SEM.

We continued to validate the effect of SF regulating the prognostic AS events in the proliferation and migration of Caco2 cells. The mRNA levels of HSPA1A and FAM50B were decreased after specific siRNA transfection in Caco2 cells (Figure 9A). As detected by the CCK8 proliferation assay, gnomically inhibiting HSPA1A or FAM50B decreased the proliferation of Caco2 significantly (Figure 9B). Wound healing and transwell assays were used to examine the effect of HSPA1A and FAM50B on the migration of Caco2 cells. It was found that both in the HSPA1Ai group, and FAM50Bi group, the Caco2 cells exhibited decreased proliferation and migration (Figures 9C,D). The results suggest that downregulation of HSPA1A and FAM50B inhibited Caco2 proliferation and migration.

## Discussion

CRC remains a leading cause of cancer-related death in the world (Siegel et al., 2020). The diagnosis and treatment of CRC have been improved dramatically, but the mortality rate continues to be high, especially in advanced patients (He et al., 2021). With sequencing technology development, new genomic markers have been proposed to guide CRC patients’ “personalized” treatment. For example, a multitude of genomic instability-associated events, including SNPs, circular RNAs, long non-coding RNA, and microRNAs, have been reported as predictors of clinical outcomes in CRC patients (Weigl et al., 2018; Ghafouri-Fard et al., 2021; Long et al., 2021).

Recently, the critical roles of AS in maintaining genomic instability have been revealed. Metastasis-associated antigen 1, an oncogenic chromatin modifier, may affect chromosomal instability by regulating related RNA splicing (Liu et al., 2020). MarcoH2A1, a replication stress-protective histone, and its alternative splicing was associated with X chromosome genomic stability (Sebastian et al., 2020b). Despite reports of recent advances in the identification of aberrant splicing events in CRC (Liu J. et al., 2018; Lian et al., 2020), there have been no relevant studies on AS signatures relative to genomic instability in CRC. Herein, we identified a group of genomic instability-related AS events in CRC and revealed their significance in predicting patient survival.

Following this line, we derived a prognostic signature based on the differential AS events. We combined single nucleotide polymorphism profiles with somatic mutation profiles of CRC and identified seven genomic instability-related AS events (PDHA1-88633-ES, KIAA1522-1632-AP, TATDN1-85088-ES, PRMT1-51042-ES, VEZT-23786-ES, AIG1-77972-AT, and PHF11-25891-AP) to construct our prognostic signature in the training set. The high-risk groups had higher UBQLN4 expression and somatic mutation count in the training set, which indicated that our prognostic signature was available to estimate genomic instability. Furthermore, two MMR genes, PMS2 and MLH3, were also higher in the high-risk group, which revalidated that our prognostic signature was associated with genomic instability. For the PMS2, Kasela, Mariann et al. found that lower expression with higher repair efficiencies (Kasela et al., 2019) indicated that the low-risk group might have better repair efficiencies. Narayanan, Sumana et al. had also found that low expression of PMS2 and MLH3 had significantly improved 5-year OS in CRC patients (Narayanan et al., 2019), which indicated that our low-risk group might have a better OS.

We proposed an ideal prognostic model and verified this model using both the test and the entire TCGA sets. This model showed a great performance in the risk stratification of CRC patients and a good potential in predicting the prognosis of CRC (AUC of the ROC curve was >0.7). Subsequently, we evaluated whether this genomic instability-related AS signature could be an independent prognostic factor. The multivariate Cox regression analysis revealed that patients with a higher risk score had poorer outcome, which was also validated in the test and the entire TCGA dataset. Finally, we established a nomogram combining the genomic instability-related AS signature with the tumor stage to enhance the convenience and accuracy of the prediction model.

Among the seven genomic instability-related AS events, PRMT1-51042-ES and VEZT-23786-ES were two positively coefficient AS events. Protein arginine methyltransferase 1 (PRMT1), the founding member of the PRMT family, has been reported to be associated with histone methylarginine and transcription activation (Yang and Bedford, 2013). Consistent with our results, previous studies have shown that a PRMT1 spice isoform could serve as a biomarker of poor prognosis in CRC (Mathioudaki et al., 2008; Yao et al., 2021). Vezatin, adherens junctions transmembrane protein (VEZT) has been identified as a tumor suppressor gene in gastric cancer (Li et al., 2015). Nevertheless, the function of VEZT variants in CRC is still unclear.

Conversely, PDHA1-88633-ES, KIAA1522-1632-AP, TATDN1-85088-ES, AIG1-77972-AT, and PHF11-25891-AP were AS events inversely correlated with the OS. Downregulation of PDHA1, a gate-keeper enzyme-linked between glycolysis and the mitochondrial citric acid cycle, has been associated with poor survival in gastric cancer and esophageal squamous cancer (Liu Z. et al., 2018; Liu et al., 2019). The KIAA1522 gene was discovered via a sequencing project of human cDNA encoding large proteins (Nagase et al., 2000). Recently, studies have indicated that KIAA1522 may act as an oncogene for non-small cell lung cell cancer (NSCLC) and breast cancer (Liu et al., 2016; Li et al., 2018). As a highly conserved nuclease, TatD DNase domain containing 1 (TATDN1), a member of the TATD family, has been found upregulated in hepatocellular carcinoma (HCC) and cisplatin-resistant NSCLC (Shen et al., 2019; Wang et al., 2019). Androgen-induced gene 1 (AIG1) is a transmembrane protein that regulates cytosolic calcium concentrations (Nickel et al., 2016). Previous studies have determined AIG1 may serve as a new biomarker for the diagnosis and prognostic evaluation of HCC and is associated with the thiopurine treatment of acute lymphoblastic leukemia (Choi et al., 2019). The deletion and methylation of PHD finger protein 11 (PHF11) were associated with chronic lymphocytic leukemia and Ewing sarcoma, respectively (Parker et al., 2011; Alholle et al., 2013). While the roles of these inversely correlated AS events in CRC remain largely unknown and require further research.

Tumor-infiltrating immune cells play essential roles in cancer development and progression. Ye et al. found that CD66b+ tumor-associated neutrophils, Tregs, and CD163+tumor-associated macrophages were significantly correlated with prognosis in CRC patients (Ye et al., 2019). In this study, we identified the differential infiltrating immune cells in CRC patients with the prognostic AS signature and found that the high-risk group showed a lower proportion of eosinophils than the low-risk group. In addition, patients with higher eosinophils had higher OS rates. Consistent with our findings, several studies have reported that eosinophil accumulation was associated with better survival in CRC patients (Pretlow et al., 1983; Harbaum et al., 2015; Prizment et al., 2016). A study of 381 primary CRC patients by Harbaum et al. found that the number of peritumoral eosinophils significantly impacted on the prognosis of CRC patients by assessing peritumoral eosinophils and intratumoral eosinophil counts (Harbaum et al., 2015). Similarly, another study involving 441 CRC patients in the United States observed that the tumor-stromal eosinophil count was an important favorable prognostic factor in CRC (Prizment et al., 2016). The anti-tumorigenic mechanisms of eosinophils in CRC include direct and indirect effects. The direct killing is achieved via degranulation and release of eosinophil-specific proteins, such as major basic protein, eosinophil cationic protein, eosinophil-derived neurotoxin, and granzymes (Legrand et al., 2010; Varricchi et al., 2018). Instead, indirect killing refers to a combination of cytokine-mediated effects, including interleukin (IL)-2, IL-5, IL-4, IL-8, and IL-17E (Benatar et al., 2010; Gatault et al., 2015). Whether our genomic instability-related AS signature could affect the anti-tumor function of eosinophil in CRC also requires further validation *in vitro* and *in vivo*. Recently, tumor immunotherapy has become a new paradigm. Inhibition of CTLA or PD-1 monoclonal antibodies is the most promising treatment approach for many cancers, including the microsatellite instability (MSI) -high advanced CRC (Messersmith, 2019). We then explored the predictive value of the prognostic AS signature in immunotherapy. The results showed that the IPS-CLTA4 score was significantly increased in the low-risk. Indeed, AS may play a ‘double-edged sword’ role in immunotherapy (Öther-Gee Pohl and Myant, 2022). Some AS variants can produce neoantigens to increase CD8^+^ T-cell immunogenicity (Smart et al., 2018). On the other hand, an alternatively spliced variant of CD19 could consult the resistance of CAR-T treatment (Sotillo et al., 2015). In this study, our prognostic AS signature had the potential ability to predict the efficacy of IPS-CLTA4 in CRC patients. Although we have no direct evidence to show that these seven screened AS events could affect the immunotherapy sensitivity, two AS events related genes, PRMT1 and PHF11, had revealed that they were associated with immune cells. PRMT1 is highly expressed in T helper cells, and the inhibition of PRMT1 could attenuate the suppressive functions of regulatory T cells (Kagoya et al., 2019). PHF11 plays an essential role in producing IgE by activated B cells (Ikari et al., 2014). These suggested that maybe AS events could affect the immunotherapy sensitivity. Further studies are required to elucidate these comprehensively.

The splicing factors (SFs) play a critical role in regulating AS events (El Marabti and Abdel-Wahab, 2021). Previous studies have demonstrated that serine and arginine-rich splicing factor 6 regulates AS to mediate CRC progression (Wan et al., 2019), and SET domain containing 2, histone lysine methyltransferase (SETD2) modulated AS events during intestinal tumorigenesis (Yuan et al., 2017). Thus, we further explored the relationship between survival-related AS events and the expression of splicing factors in CRC. Eight SFs, including SNRPN, HSPA1A, HSPB1, BAG2, BCAS1, DDX3Y, MSI1, and FAM50B, were associated with survival-related AS events. Among these SFs, HSPA1A and FAM50B were associated with OS in CRC. We found that patients with lower HSPA1A expression levels had higher OS rates, which was consistent with previous studies (Xing et al., 2021). As a member of the heat shock proteins (HSPs) family, heat shock protein family A (Hsp70) member 1A (HSPA1A) exerted cytoprotective and immunological functions (Wang et al., 2021). Recently, Huang et al. (2021) found that HSPA1A could regulate two types of AS events (SNX5-58744-AT and SNX5-587745-AT), which were correlated with distant metastasis, through the “Class Ⅰ MHC mediated antigen processing and presentation” pathway in mesothelioma. Our study also found that patients with lower FAM50B expression levels also had higher OS. Loss of FAM50B (also known as Family with sequence similarity 50, member B) expression has also been identified in almost 4% of cancers in the TCGA database. Silencing FAM50B can reduce cellular fitness and cause apoptosis and dysregulation of transcription (Thompson et al., 2021). It has been validated that as a splicing factor, FAM50B serves an independent prognostic factor in glioblastoma (Qiu et al., 2021). In order to assess the impact of HSPA1A and FAM50B in the progression of CRC, we performed functional analysis on Caco2 cells. The results showed that both individual and simultaneous HSPA1A and FAM50B knockdown showed proliferation and migration inhibition function.

Though our study provides significant insights to explore the relationship between genome instability and CRC patients’ prognosis, some limitations should also be considered. First, as the TCGA database is the only available database providing the alternative splicing events data, an external examination is unpracticable. In the future, we hope an external examination could be conducted. Second, additional studies will be necessary to unravel the biological roles of these AS events *in vivo* and *in vitro*.

In conclusion, we developed and validated a risk prognostic signature comprising seven genomic instability-related AS events, which could serve as an independent prognostic biomarker for the survival of CRC patients and reflect the change in the microenvironment of CRC. HSPA1A and FAM50B play an important role in the proliferation and migration of Caco2 cells. Our data suggest that this genomic instability-related AS signature and its regulatory network may have important implications for developing new therapeutic targets and individualized therapy in patients with CRC.

## Data Availability

The datasets presented in this study can be found in online repositories. The names of the repository/repositories and accession number(s) can be found in the article/Supplementary Material.

## Glossary

AS: Alternative splicing
AD: Alternate Donor site
AA: Alternate Acceptor site
AT: Alternate Terminator
AP: Alternate Promoter
AUC: Area under curve
AIG1: Androgen-induced gene 1
CRC: Colorectal cancer;
ES: Exon Skip
GU: Genomic unstable
GS: Genomic stable
HCC: Hepatocellular carcinoma
HR: Hazard ratio
HSPA1A: Heat shock protein family A (Hsp 70) member 1A
LASSO: Least absolute shrinkage and selection operator
MMR: Mismatch repair
ME: Mutually Exclusive Exons
NSCLC: Non-small cell lung cell cancer
OS: Overall survival
PXR: Pregnane X receptor
PSI: Percent Splicing index
POLE: Polymerase ε
PRMT1: Protein arginine methyltransferase 1
PHF11: PHD finger protein 11
RI: Retained Intron
ROC: Receiver operating characteristic
SAM: Significance analysis of microarrays
SNP: Single nucleotide polymorphism
SF: Splicing factors
SETD2: SET domain containing 2, histone lysine methyltransferase
TATDN1: TatD DNase domain containing 1
TCGA: The Cancer Genome Altas
UGT1A: UDP glucuronosyltransferase family 1 member A complex locus
VEZT: Vezatin, adherens junctions transmembrane protein.
